# Identification of the Ovine Keratin-Associated Protein 22-1 (KAP22-1) Gene and Its Effect on Wool Traits

**DOI:** 10.3390/genes8010027

**Published:** 2017-01-11

**Authors:** Shaobin Li, Huitong Zhou, Hua Gong, Fangfang Zhao, Jiqing Wang, Xiu Liu, Yuzhu Luo, Jon G. H. Hickford

**Affiliations:** 1 Gansu Key Laboratory of Herbivorous Animal Biotechnology, Faculty of Animal Science and Technology, Gansu Agricultural University, Lanzhou 730070, China; lisb2008@hotmail.com (S.L.); Zhou@lincoln.ac.nz (H.Z.); zhaoFangfang@gsau.edu.cn (F.Z.); wangjq@gsau.edu.cn (J.W.); liuxiu@gsau.edu.cn (X.L.); 2 International Wool Research Institute, Gansu Agricultural University, Lanzhou 730070, China; Hua.Gong@lincolnuni.ac.nz; 3 Gene-marker Laboratory, Faculty of Agricultural and Life Sciences, Lincoln University, Lincoln 7647, New Zealand

**Keywords:** Keratin-associated protein KAP22-1, variation, mean fiber curvature (MFC), wool yield, sheep

## Abstract

Keratin-associated proteins (KAPs) are structural components of wool and hair fibers. To date, eight high glycine/tyrosine KAP (HGT-KAP) families have been identified in humans, but only three have been identified in sheep. In this study, the putative ovine homolog of the human KAP22-1 gene (*KRTAP22-1*) was amplified using primers designed based on a human *KRTAP22-1* sequence. Polymerase chain reaction-single stranded conformational polymorphism (PCR-SSCP) was used to screen for variation in *KRTAP22-1* in 390 Merino × Southdown-cross lambs and 75 New Zealand (NZ) Romney sheep. Three PCR-SSCP banding patterns were detected and DNA sequencing revealed that the banding patterns represented three different nucleotide sequences (*A–C*). Two single nucleotide polymorphisms (SNPs) were identified in these sequences. Variant *B* was most common with a frequency of 81.3% in NZ Romney sheep, while in the Merino × Southdown-cross lambs, *A* was more common with a frequency of 51.8%. The presence of *B* was found to be associated with increased wool yield and decreased mean fiber curvature (MFC). Sheep of genotype *BB* or *AB* had a higher wool yield than those of genotype *AA*. These results suggest that ovine *KRTAP22-1* variation may be useful when developing breeding programs based on increasing wool yield, or decreasing wool curvature.

## 1. Introduction

Keratin-associated proteins (KAPs) and keratins are the main structural proteins of wool and hair fibers. The former create a semi-rigid matrix with the keratin intermediate filaments (IFs) [1] and they play an important role in defining the physico-mechanical properties of the fibers. KAPs are a complex class of proteins and typically possess a high cysteine content. The KAPs have been classified into three broad groups according to their amino acid composition: the high sulfur (HS; ≤30 mol% cysteine), the ultra-high sulfur (UHS; >30 mol% cysteine) and the high glycine-tyrosine (HGT; 35–60 mol% glycine and tyrosine) groups [2]. More than 100 KAP genes have been identified across species and they have been divided into 27 KAP families [3]. Of these KAP families: 1–3, 10–16 and 23–27 are HS-KAPs; 4, 5, 9 and 17 are UHS-KAPs and 6–8 and 18–22 are HGT-KAPs [3,4].

Wool varies in HGT-KAP content ranging from less than 1% in Lincoln wool, to between 4% and 12% in Merino wool [5]. The wide range in the proportional content of HGT-KAPs in different wools and the extensive variation in the genes for the HGT-KAP genes [6,7,8] suggests that the HGT-KAPs may have important function in the wool fiber.

To date, three HGT-KAP gene families have been reported in sheep: KAP6, KAP7 and KAP8. There has been no report of the presence of other HGT-KAPs. The KAP22-1 gene (*KRTAP22-1*) has been identified in humans [9], but it has not been described in sheep. In this study, we describe the identification of a sequence encoding the putative ovine *KRTAP22-1*, report variation in this gene detected using polymerase chain reaction-single stranded conformational polymorphism (PCR-SSCP), and reveal associations between this genetic variation and variation in wool traits.

## 2. Materials and Methods

### 2.1. Sheep Blood and Wool Samples

Three hundred ninety lambs, produced over three years from crosses of Merino ewes × Southdown (n = 4; 188, 75, 59 and 68 progeny per ram), and seventy-five New Zealand (NZ) Romney lambs (n = 75, sourced from five farms) were used to search for variation in *KRTAP22-1*. The 390 Merino × Southdown lambs were subsequently used for the association study. Blood samples from all these sheep were collected onto FTA cards (Whatman BioScience, Middlesex, UK) and genomic DNA was purified using a two-step procedure described by Zhou et al. [10].

Wool samples were collected at 12 months of age (first shearing) from the mid-side of the Merino × Southdown-cross lambs. Greasy fleece weight (GFW) was measured at shearing, and other wool traits were measured by the New Zealand Wool Testing Authority Ltd (Ahuriri, Napier, NZ), including mean fiber diameter (MFD), fiber diameter standard deviation (FDSD), coefficient of variation of fiber diameter (CVFD), mean staple length (MSL), mean fiber curvature (MFC), mean staple strength (MSS) and prickle factor (PF). Wool yield (%) was measured and used to calculate the clean fleece weight (CFW).

### 2.2. Search for an Ovine Homolog of the Human KRTAP22-1 Gene in the Sheep Genome Sequence

The coding sequence of a human *KRTAP22-1* sequence (AP001708) was used to BLAST search the Ovine Genome Assembly v4.0 (http://blast.ncbi.nlm.nih.gov/Blast.cgi). The genome sequences that showed highest homology with the human *KRTAP22-1* sequence were presumed to be ovine *KRTAP22-1* sequences. These sequences were used to design PCR primers for amplifying the entire coding region of this gene from sheep genomic DNA.

### 2.3. PCR Primers and Amplification of Sheep Genomic DNA

The sequences of the PCR primers designed were: 5′-TATGAGTGCAACAGTGACTG-3′ and 5′-CCATGTTTTGAATAGACAAGC-3′. They were synthesized by Integrated DNA Technologies (Coralville, IA, USA). PCR amplification was performed in a 15-μL reaction containing the genomic DNA on one 1.2-mm punch of FTA paper, 0.25 μM of each primer, 150 μM of each dNTP (Bioline, London, UK), 2.5 mM of Mg^2+^, 0.5 U of *Taq* DNA polymerase (Qiagen, Hilden, Germany) and 1× reaction buffer supplied with the enzyme. The thermal profile consisted of 2 min at 94 °C, followed by 35 cycles of 30 s at 94 °C, 30 s at 61 °C and 30 s at 72 °C, with a final extension of 5 min at 72 °C. Amplification was carried out in S1000 thermal cyclers (Bio-Rad, Hercules, CA, USA).

Amplicons were visualized by electrophoresis in 1% agarose gels (Quantum Scientific, Queensland, Australia), using 1 × TBE buffer (89 mM Tris, 89 mM boric acid, 2 mM Na_2_EDTA) containing 200 ng/mL of ethidium bromide.

### 2.4. Screening for Variation in KRTAP22-1

The PCR amplicons were screened for sequence variation using SSCP analysis. A 0.7-μL aliquot of each amplicon was mixed with 7 μL of loading dye (98% formamide, 10 mM EDTA, 0.025% bromophenol blue, 0.025% xylene-cyanol). After denaturation at 95 °C for 5 min, the samples were rapidly cooled on wet ice and then loaded on 16 cm × 18 cm, 14% acrylamide: bisacrylamide (37.5:1) (Bio-Rad) gels. Electrophoresis was performed using Protean II xi cells (Bio-Rad) in 0.5× TBE buffer, under the electrophoretic conditions 18 °C, 300 V for 16 h. Gels were silver-stained according to the method of Byun et al. [11].

### 2.5. Sequencing of Allelic Variants and Sequence Analysis

PCR amplicons representing different banding patterns from sheep that appeared to be homozygous were sequenced in both directions at the Lincoln University DNA sequencing facility, New Zealand. Alleles that were only found in heterozygous sheep were sequenced using an approach described by Gong et al. [12]. Briefly, a band corresponding to the allele was excised as a gel slice form the polyacrylamide gel, macerated, and then used as a template for re-amplification with the original primers. This second amplicon was then sequenced. Sequence alignments, translations and phylogenetic analysis were carried out using DNAMAN (version 5.2.10, Lynnon BioSoft, Vaudreuil, Canada). Phylogenetic tree was constructed using Observed Divergency method with 1000 bootstrap trials based on the predicated amino acid sequence.

### 2.6. Statistical Analyses

Statistical analyses were performed using Minitab version 16 (Minitab Inc., State College, PA, USA). General linear models (GLMs) were used to assess the effect of the presence/absence of the *KRTAP22-1* variants on various wool traits for the 390 Merino × Southdown lambs. For genotypes with a frequency >5% (and thus that had an adequate sample size), GLMs were used to compare the various wool traits among these genotypes and with a Bonferroni correction being applied to reduce the chances of obtaining false positive results during the multiple comparisons. Sire was found to affect (*p* < 0.05) all the wool traits and was included in the models as a random factor. Gender was found to affect (*p* < 0.05), or potentially affect (*p* < 0.20), wool traits, and was therefore fitted as a fixed factor into the models. Birth rank was not found to affect or potentially affect wool traits, and was not factored into the models.

## 3. Results

### 3.1. Identification of KRTAP22-1 in the Sheep Genome

**Figure 1:**
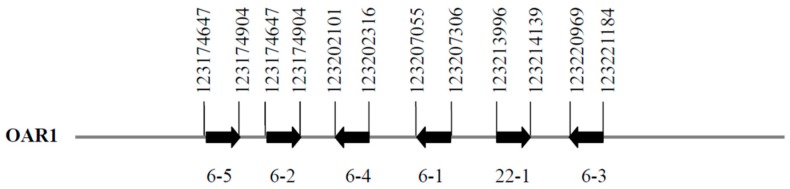
Location of sheep genome region that is homologous to *KRTAP22-1*, together with five other previously identified *KRTAPs* on sheep chromosome 1. Horizontal arrow bars represent the coding regions of *KRTAPs* and the arrowheads indicate the direction of transcription. The numbers below the horizontal arrow bars indicate the name of the respective KAP gene (e.g., 6-5 represents *KRTAP6-5*). The nucleotide positions refer to NC_019458.2.

A BLAST search of the Ovine Genome Assembly v4.0 using the human *KRTAP22-1* coding sequence (AP001708) revealed a homologous region on sheep chromosome 1. Analysis of the sequence in this homologous region led to the identification of a 144-bp open reading frame at OAR1: 123213996–123214139. Five previously identified ovine KAP genes were also found near this open reading frame and these from centromere to telomere were *KRTAP6-1*, *KRTAP6-3*, *KRTAP6-4*, *KRTAP6-2* and *KRTAP6-5* (Figure 1).

**Figure 2:**
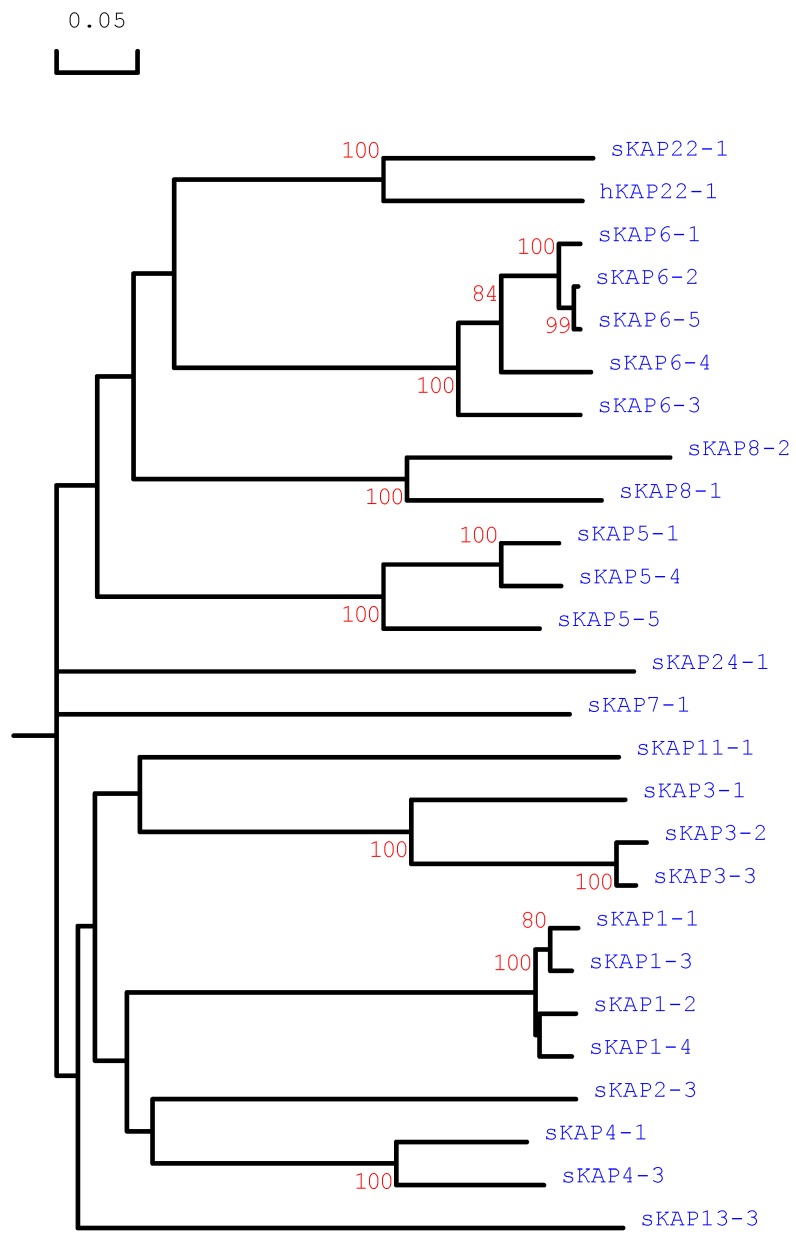
Phylogenetic tree of the sheep genomic regions identified, together with human KAP22-1. The tree was constructed using the predicted amino acid sequences. The ovine KAPs are indicated with the prefix “s” and the human KAP has the prefix “h”. The numbers at the forks indicate the bootstrap confidence values and only those equal to or higher than 70% are shown. The GenBank accession numbers for the human KAP22-1 is AP001708. The GenBank accession numbers for the sheep KAPs are X01610, HQ897973, X02925, X01610, U60024, M21099, M21100, M21103, X73462, EU239778, X55294, X73434, X73435, M95719, KT725827, KT725833, KT725838, KT725841, X05638, X05639, KF220646, HQ595347, JN377429 and JX112014 (for sKAP1-1, sKAP1-2, sKAP1-3, sKAP1-4, sKAP2-3, sKAP3-1, sKAP3-2, sKAP3-3, sKAP4-1, sKAP4-3, sKAP5-1, sKAP5-4, sKAP5-5, sKAP6-1, sKAP6-2, sKAP6-3, sKAP6-4, sKAP6-5, sKAP7-1, sKAP8-1, sKAP8-2, sKAP11-1, sKAP13-3 and sKAP24-1, respectively).

The open reading frames identified were translated into amino acid sequences, and sequence comparison with known sheep KAPs together with human KAP22-1, revealed that this region was clustered with the human KAP22-1 sequence and formed a group that is distinct to other sheep KAP families (Figure 2). It suggested the presence of sheep *KRTAP22-1*.

### 3.2. Detection of Variation in Ovine KRTAP22-1

**Figure 3:**
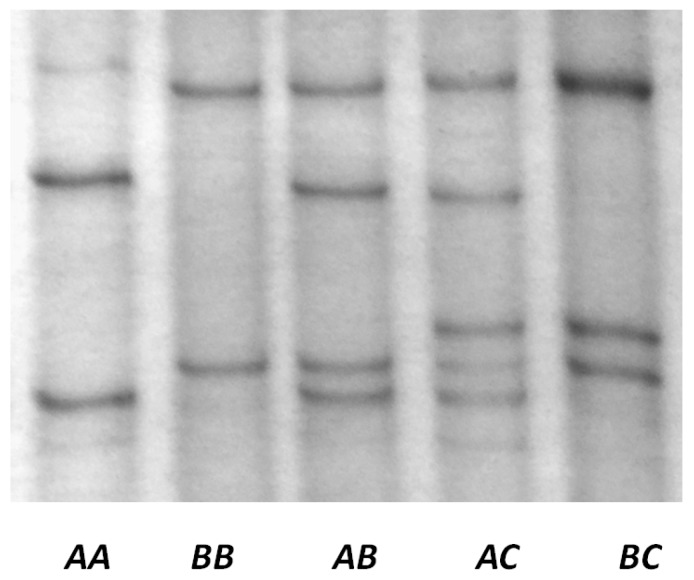
PCR–single-stranded conformational polymorphism (PCR–SSCP) of the ovine KAP22-1 gene.

There were three PCR-SSCP banding patterns detected for ovine *KRTAP22-1*, with either one or a combination of two banding patterns observed for each sheep (Figure 3). DNA sequencing revealed that these PCR-SSCP patterns represented three different nucleotide sequences (*A, B* and *C*) (Table 1). These sequences have been deposited into GenBank with accession numbers KX377616, KX377617 and KX377618. Two single nucleotide polymorphisms (SNPs) were identified among the three sequences. One SNP was located 28 bp upstream of the nominal TATA box sequence and the other SNP was located in the coding region. The coding region SNP was synonymous.

### 3.3. Amino Acid Composition of Ovine KAP22-1

The ovine *KRTAP22-1* sequences would encode a polypeptide of 47 amino acid residues. This would have a high content of glycine and tyrosine (51.1 mol%), and moderate levels of cysteine (14.9 mol%) and serine (8.3 mol%). The putative KAP22-1 protein would therefore be basic, with a predicted isoeletric point (pI) value of 7.65.

### 3.4. Genotypes and Allele Frequencies in NZ Romney and Merino-Cross Sheep

Five genotypes were detected in Merino × Southdown-cross lambs, and they were as follows: *AA*, *AB*, *AC*, *BB* and *BC*. Genotypes *AC* and *BC* were not detected in the NZ Romney sheep.

The frequencies of the *KRTAP22-1* variants in the NZ Romney sheep were: *A*: 18.7 % and *B*: 81.3%; while those in Merino × Southdown-cross lambs were: *A*: 51.8%, *B*: 47.2% and *C*: 1.0%. Variant *B* was very common in NZ Romney lambs, while, in Merino × Southdown-cross lambs, *A* was more common. The four sires of the Merino × Southdown-cross lambs were all of genotype *AB*.

### 3.5. Effect of Variation in KRTAP22-1 on Wool Traits

Of the three variants detected in Merino × Southdown-cross lambs, variant *C* occurred at a very low frequency (<5%) and accordingly its association with wool traits was not analyzed (sheep with these genotypes were discarded from the genotype analyses). In the presence/absence models, the presence of *B* was found to be associated with increased wool yield and decreased MFC (Table 2).

With three genotypes (*AA*, *BB* and *AB*) that occurred at a frequency >5% in Merino × Southdown-cross lambs, an effect of genotype was detected for wool yield (Table 3). Sheep of genotype *AB* or *BB* had a higher wool yield than those of genotype *AA*.

## 4. Discussion

This study describes the identification of a new ovine HGT-KAP. The putative ovine *KRTAP22-1* was clustered with several previously described KAP genes and displayed a lower sequence similarity to any known ovine KAP gene, when compared to *KRTAP22-1* from humans. This gene was located between *KRTAP* 6-1 and *KRTAP* 6-3, and this is consistent with the location of human *KRTAP22-1* [9]. This suggests that the gene represents the ovine *KRTAP22-1* sequence. The identification of ovine *KRTAP22-1* brings the total number of HGT-KAPs identified in sheep, from eight to nine.

The peptide encoded for by the ovine *KRTAP22-1* sequence was predicted to contain 47 amino acids and more than half of these amino acids were glycine and tyrosine. This is consistent with other HGT-KAPs; however, the number of repeated occurrences of glycine-tyrosine and glycine-tyrosine-glycine in KAP22-1 was small when compared to other ovine HGT-KAPs (KAP6–KAP8). Until now, nearly all the HGT-KAPs are basic (except KAP8-2) [2], and this was also the case for KAP22-1.

Two SNPs were detected in ovine *KRTAP22-1* and these produced three unique variant sequences. Both of the SNPs were T/C transitions. The coding region SNP was synonymous. Among the three variants, *B* was very common in Romney sheep, while in Merino × Southdown-cross lambs *A* was more common.

Variation in *KRTAP22-1* was found to be associated with two wool traits, MFC and wool yield. However, the most enduring effect by *KRTAP22-1* appeared to be on the wool yield, for which a sizeable difference in mean among common genotypes was detected and a difference that reinforced the conclusions drawn from the variant absence/presence models. Sheep with *B* have a higher wool yield, but a lower MFC. This is consistent with the correlation that has been reported between these two traits, with a moderate negative correlation (0.3 < |r| ≤ 0.7) found between MFC and wool yield (r = −0.518) [13]. The wool yield for the Merino × Southdown-cross lambs was lower at 73% (Table 3), than in Romney sheep (80%) and this is consistent with other reports [14].

Lambs with *B* had lower fiber curvature. The correlation between MFC and fiber crimp has been reported to be high at 0.85 [15], so curvature is a reasonable proxy for the crimp of fiber. “Low-curvature” wool generally has a curvature less than 50°/mm, while “medium curvature” wool is from 60 to 90°/mm and “high curvature” wool is greater than 100°/mm, this being associated typically with a high crimp frequency. The Merino × Southdown-cross lambs in this study had a medium curvature wool (Table 3), while the Romney sheep had low-curvature wool (38°/mm) [16].

The results are consistent with the predicted function of HGT-KAPs. It has been reported that the content of HGT-KAPs is decreased in Merino felting luster (FL) mutant wool that loses crimp [17], and that the helical angle of IFs in the orthocortex is associated with fiber curvature [18]. The HGT-KAPs are predominantly present in the orthocortex and are thought to have some associations with the crimp of wool fiber [19]. The results from this research confirm that variation in *KRTAP22-1* may affect those traits.

Although the variation in the SNP in the coding region of *KARTAP22-1* is synonymous, and would not result in any amino acid substitution, it may affect the expression or structure of the protein. It has been reported that silent mutations may affect mRNA translation rates and thus potentially change the way that protein folds [20]. It is also possible that the effects observed for *KRTAP22-1* are be due to its linkage to other *KRTAPs* or *KRTs* on the same chromosome. The location of *KRTAP22-1* is interesting in that it is found within KAP6 family members and the biological significance of this needs more study.

## 5. Conclusions

These findings confirm that ovine *KRTAP22-1* is variable and suggests that variation in the gene may need to be considered when developing breeding programs based on improving wool curvature or wool yield.

## Figures and Tables

**Table 1:** SNPs and alleles of the ovine *KRTAP22-1*.

SNP	Allele			Amino Acid Change
	*A*	*B*	*C*	
C.-100C/T	C	C	T	5′UTR
C.45T/C	T	C	C	No change

**Table 2:** Association of *KRTAP22-1* variants with various wool traits.

Trait ^1^	Variant	n		Mean ± SE ^2^		*p* ^3^
		Absent	Present	Absent	Present	
GFW (kg)	*A*	79	311	2.30 ± 0.07	2.33 ± 0.06	0.628
	*B*	99	291	2.37 ± 0.07	2.31 ± 0.06	0.191
CFW (kg)	*A*	79	311	1.69 ± 0.06	1.69 ± 0.05	0.931
	*B*	99	291	1.70 ± 0.05	1.69 ± 0.05	0.844
Yield (%)	*A*	79	311	72.9 ± 0.99	72.0 ± 0.79	0.244
	*B*	99	291	70.8 ± 0.91	72.6 ± 0.79	**0.008**
MFD (µm)	*A*	79	311	19.6 ± 0.31	19.5 ± 0.25	0.705
	*B*	99	291	19.4 ± 0.30	19.6 ± 0.25	0.547
FDSD (µm)	*A*	79	311	4.28 ± 0.11	4.16 ± 0.09	0.139
	*B*	99	291	4.17 ± 0.10	4.19 ± 0.30	0.828
CVFD (%)	*A*	79	311	22.0 ± 0.36	21.7 ± 0.30	0.281
	*B*	99	291	21.9 ± 0.35	21.7 ± 0.30	0.503
MSL (mm)	*A*	79	311	84.2 ± 1.99	84.6 ± 1.63	0.796
	*B*	99	291	85.5 ± 1.93	84.3 ± 1.62	0.368
MSS (N/ktex)	*A*	79	311	21.1 ± 1.25	23.4 ± 1.02	0.192
	*B*	99	291	21.7 ± 1.21	22.2 ± 1.02	0.526
MFC (^o^/mm)	*A*	79	311	86.5 ± 2.43	89.4 ± 1.98	0.128
	*B*	99	291	91.6 ± 2.34	87.9 ± 1.97	**0.032**
PF (%)	*A*	79	311	2.12 ± 0.36	2.05 ± 0.29	0.816
	*B*	99	291	2.13 ± 0.35	2.05 ± 0.28	0.762

^1^ GFW—Greasy Fleece Weight; CFW—Clean Fleece Weight; MFD—Mean Fiber Diameter; FDSD—Fiber Diameter Standard Deviation; CVFD—Coefficient of Variation of Fiber Diameter; MSL—Mean Staple Length; MSS—Mean Staple Strength; MFC—Mean Fiber Curvature; PF—Prickle Factor (percentage of fibers over 30 µm). ^2^ Predicted marginal means and standard errors derived from GLMs with variant absent/ present, sire (random effect) and gender (fixed effect) being factored into the models. ^3^ *p* < 0.05 are in bold.

**Table 3:** The effect of *KRTAP22-1* genotype on various wool traits.

Trait ^1^	Mean ± SE ^2^			*p*
	*AA* (n = 93)	*AB* (n = 212)	*BB* (n = 77)	
GFW (kg)	2.38 ± 0.07	2.31 ± 0.06	2.29 ± 0.07	0.341
CFW (kg)	1.71 ± 0.06	1.69 ± 0.05	1.70 ± 0.06	0.918
Yield (%)	70.9 ± 0.97 ^b^	72.6 ± 0.83 ^a^	73.0 ± 1.00 ^a^	**0.044**
MFD (µm)	19.5 ± 0.31	19.6 ± 0.27	19.6 ± 0.32	0.838
FDSD (µm)	4.20 ± 0.10	4.16 ± 0.09	4.27 ± 0.11	0.385
CVFD (%)	21.9 ± 0.36	21.6 ± 0.31	21.9 ± 0.37	0.321
MSL (mm)	85.9 ± 1.98	84.1 ± 1.71	84.1 ± 2.03	0.488
MSS (N/ktex)	21.8 ± 1.24	22.7 ± 1.08	21.2 ± 1.28	0.306
MFC (^o^/mm)	91.7 ± 2.45	89.0 ± 2.12	86.8 ± 2.51	0.120
PF (%)	2.46 ± 0.53	2.45 ± 0.46	3.04 ± 0.55	0.387

^1^ GFW—Greasy Fleece Weight; CFW—Clean Fleece Weight; MFD—Mean Fiber Diameter; FDSD—Fiber Diameter Standard Deviation; CVFD—Coefficient of Variation of Fiber Diameter; MSL—Mean Staple Length; MSS—Mean Staple Strength; MFC—Mean Fiber Curvature; PF—Prickle Factor (percentage of fibers over 30 µm). ^2^ Predicted marginal means and standard errors derived from the GLMs with genotype, sire (random effect) and gender (fixed effect) being factored into the models with a Bonferroni correction to adjust for repetitive testing. Means within rows that do not share a superscript letter are significantly (*p* < 0.05) different and bolded.
